# Case Report: Complete endoscopic submucosal dissection for occult superficial esophageal squamous cell carcinoma concealed by a large esophageal leiomyoma

**DOI:** 10.3389/fsurg.2026.1819413

**Published:** 2026-05-20

**Authors:** Xiaoying Yang, Yaowen Hu, Wangyang Chen, Shengyu Zhang, Yonglin Wang, Kun He, Qingwei Jiang, Dong Wu

**Affiliations:** 1 State Key Laboratory of Complex Severe and Rare Diseases, Department of Gastroenterology, Peking Union Medical College Hospital, Chinese Academy of Medical Sciences & Peking Union Medical College, Beijing, China; 2 Department of Gastroenterology, Zhejiang Provincial People’s Hospital, Hangzhou Medical College, Hangzhou, Zhejiang Province, China

**Keywords:** case report, diagnostic pitfall, endoscopic submucosal dissection, esophageal squamous cell carcinoma, leiomyoma

## Abstract

**Introduction:**

Benign-appearing gastrointestinal lesions may occasionally conceal malignancy and pose a diagnostic challenge. Esophageal leiomyomas are benign neoplasms; however, careful mucosal assessment remains essential to avoid missed diagnoses.

**Case description:**

We report the case of a 53-year-old asymptomatic woman in whom a large esophageal leiomyoma masked a superficial esophageal squamous cell carcinoma (ESCC). Subtle mucosal irregularity detected on endoscopy prompted further evaluation with narrow-band imaging and targeted biopsies, confirming carcinoma *in situ*. Following multidisciplinary discussion and shared decision-making that incorporated patient preferences, selective endoscopic submucosal dissection (ESD) was performed to achieve complete resection of the carcinoma while preserving the underlying leiomyoma.

**Conclusion:**

En bloc resection was achieved without complications. Histopathology confirmed a well-differentiated superficial ESCC with submucosal invasion (>200 μm, pT1b), negative horizontal and vertical margins, and no lymphovascular invasion. After postoperative pathology review, additional surgical treatment was recommended because of the risk associated with submucosal invasion; however, the patient strongly declined surgery. Contrast-enhanced CT showed no evidence of regional lymph node metastasis, and the patient subsequently underwent radiotherapy at a local hospital. At 2-year follow-up, endoscopic re-examination showed no recurrence, and the leiomyoma remained stable in size. This case underscores the importance of careful evaluation for occult malignancy hidden behind benign-appearing lesions and demonstrates that favorable outcomes can be achieved through individualized, multidisciplinary management, including endoscopic resection, adjuvant therapy, and close surveillance.

## 1 Introduction

Esophageal squamous cell carcinoma (SCC) and esophageal leiomyomas are distinct pathological entities with different cellular origins and clinical implications. SCC arises from the esophageal mucosal epithelium and remains one of the most prevalent histological subtypes of esophageal malignancy worldwide, whereas leiomyomas are benign smooth muscle tumours that typically originate from the submucosa or muscularis propria. Leiomyomas are often asymptomatic and are frequently discovered incidentally during endoscopic or radiological examinations performed for unrelated indications.

Although each condition is relatively common in isolation, the coexistence of SCC and leiomyoma within the same esophageal segment is exceedingly rare, with only sporadic cases reported in the literature (1). This rarity contributes to a potential diagnostic pitfall, as the presence of a benign-appearing submucosal lesion may dominate the endoscopic appearance and distract attention from subtle mucosal abnormalities. Consequently, early malignant changes arising on or adjacent to a leiomyoma may be overlooked, particularly in asymptomatic patients undergoing routine screening.

Benign-appearing esophageal lesions should therefore not be dismissed without careful evaluation. Even minimal mucosal irregularity warrants further assessment, as it may conceal an underlying malignancy. Early and comprehensive diagnostic work-up, including the use of advanced endoscopic imaging techniques, is essential to ensure timely diagnosis and appropriate management (2). Failure to recognise such occult malignancy may delay treatment and adversely affect patient outcomes.

The coexistence of superficial esophageal SCC and leiomyoma presents unique challenges in both diagnosis and treatment. Clinicians must balance oncological safety with the preservation of esophageal structure and function, particularly in patients without symptoms. In this report, we describe the successful management of a patient with coexisting superficial esophageal SCC and leiomyoma using selective endoscopic submucosal dissection (ESD). This case highlights the diagnostic challenges posed by benign-appearing lesions masking malignancy and illustrates the role of minimally invasive, patient-centred strategies in achieving optimal clinical outcomes.

## 2 Case presentation

A 53-year-old woman presented to our hospital for routine upper gastrointestinal endoscopy as part of a regular health screening programme. She was entirely asymptomatic and denied dysphagia, odynophagia, retrosternal discomfort, gastroesophageal reflux, regurgitation, or unintentional weight loss. Her past medical history was unremarkable, and she had no known risk factors for esophageal malignancy. There was no relevant family history.

Prior to referral, a thoracic computed tomography (CT) scan performed at a local hospital had incidentally identified a well-defined submucosal mass measuring approximately 4.4 × 2.5 cm in the mid-esophagus. The imaging characteristics were suggestive of a benign mesenchymal tumour, most likely a leiomyoma. She was therefore referred to our tertiary centre for further diagnostic evaluation and management.

On admission, upper gastrointestinal endoscopy demonstrated a prominent submucosal bulge extending from 28 to 33 cm from the incisors. Although the majority of the overlying mucosa appeared smooth and intact, careful inspection revealed a focal area of surface roughening on the anal side of the lesion. This subtle mucosal abnormality raised suspicion of a possible coexisting epithelial lesion rather than a purely benign submucosal process.

**Figure 1:**
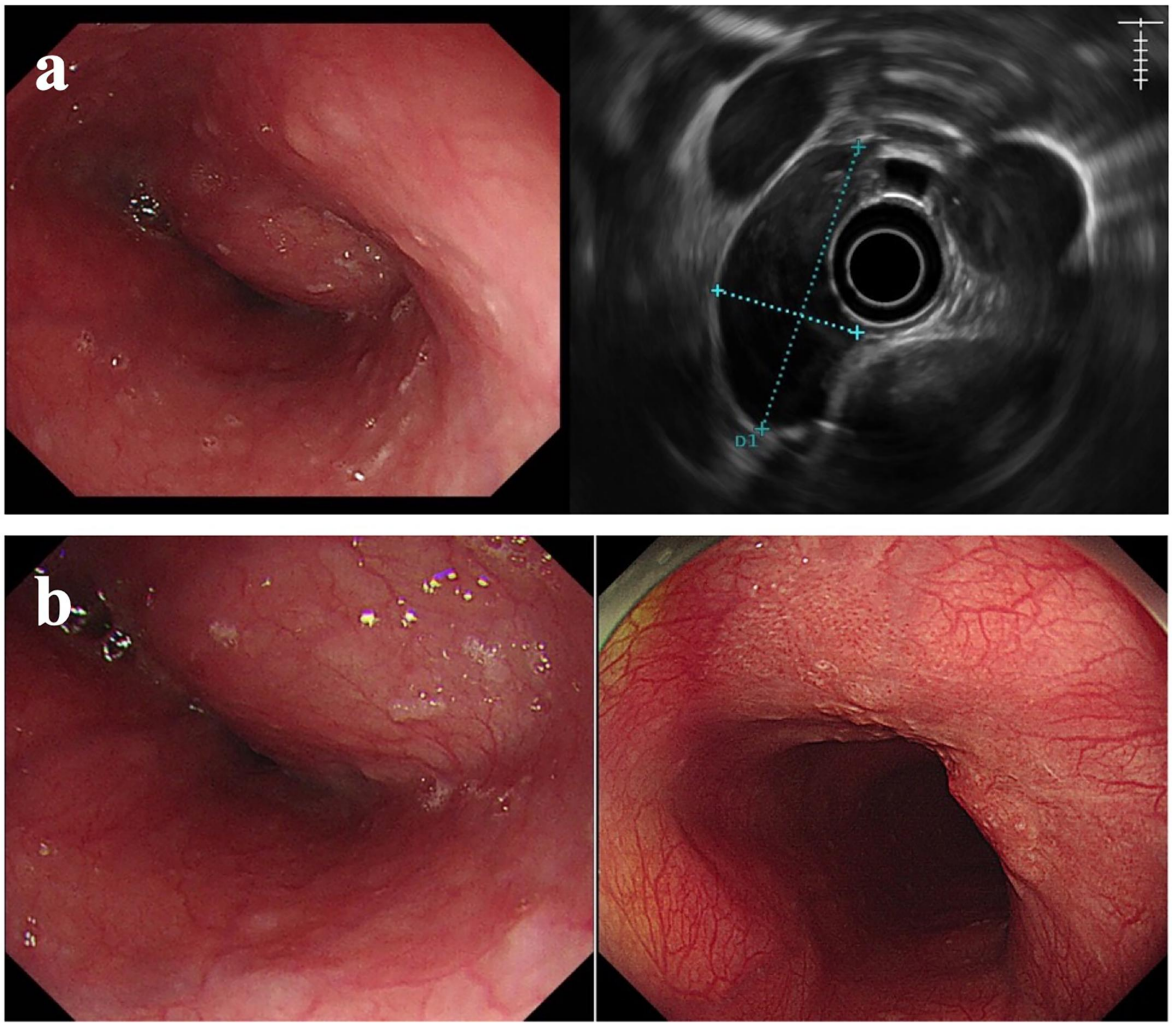
Endoscopic appearance of the esophageal lesion. **(a)** Endoscopic view showing a large submucosal protrusion in the mid-esophagus. The oral side appears smooth, while focal surface irregularity is noted on the anal side. **(b)** Submucosal protrusion with distinct surfaces. Smooth on the oral side and rough on the anal side, resembling a type 0-IIa + IIc lesion measuring 2.0 × 2.0 cm. Esophageal leiomyoma was highly suspected.

Endoscopic ultrasonography (EUS) was subsequently performed to further characterise the lesion. EUS revealed a well-circumscribed hypoechoic mass arising from the muscularis propria layer, consistent with an esophageal leiomyoma, measuring 4.4 × 2.5 cm. Notably, focal thickening of the overlying mucosal layer was observed, corresponding to the area of surface irregularity seen on conventional endoscopy (Figure 1).

**Figure 2:**
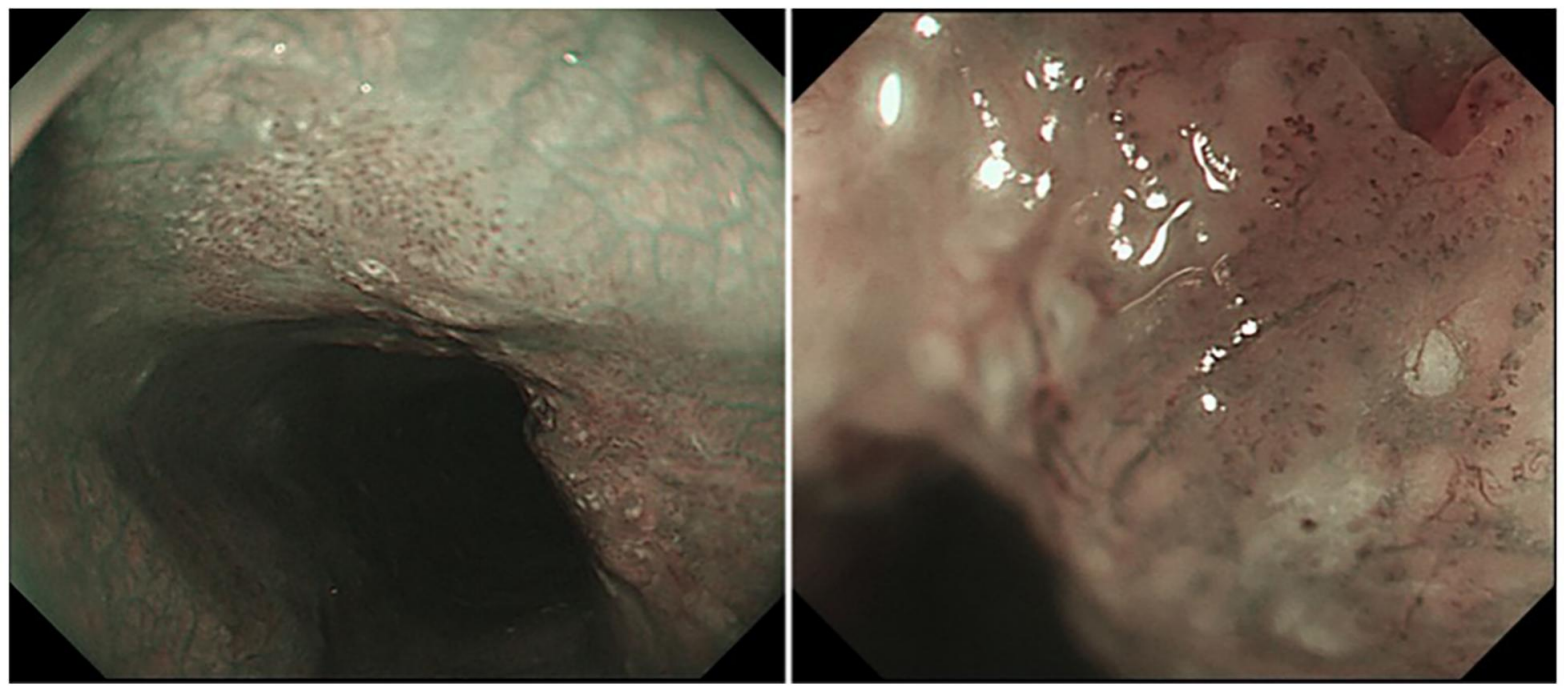
Magnifying endoscopy showing irregular IPCL patterns. Further magnifying endoscopy revealed type B1 based on the AB classification of intrapapillary capillary loops (IPCL), consistent with esophageal squamous cell carcinoma.

Given these findings, advanced endoscopic imaging was undertaken. Narrow-band imaging (NBI) with magnification revealed an irregular intrapapillary capillary loop (IPCL) pattern over the roughened mucosal area, suggestive of early neoplastic change. Lugol’s iodine chromoendoscopy further demonstrated a well-demarcated iodine-unstained region, reinforcing concern for malignant transformation (Figure 2). Targeted biopsies obtained from this area confirmed the diagnosis of squamous cell carcinoma *in situ*.

Comprehensive staging investigations were subsequently performed. Contrast-enhanced CT imaging of the chest and abdomen, along with cervical ultrasonography, revealed no evidence of regional lymphadenopathy or distant metastasis. The lesion was therefore staged as an early, superficial esophageal carcinoma.

**Figure 3:**
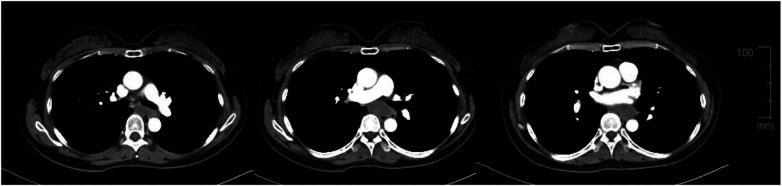
Preoperative contrast-enhanced CT showing the anatomical relationship of the leiomyoma. The image demonstrates that the esophageal leiomyoma is closely adjacent to the heart and major vessels, highlighting the high risk of major intraoperative bleeding and potential technical challenges for resection.

Management options were discussed in detail within a multidisciplinary team setting. Surgical resection of both the carcinoma and the underlying leiomyoma was considered; however, the patient expressed significant concerns regarding the potential morbidity of surgery, including postoperative reflux and its impact on long-term quality of life. In addition, preoperative contrast-enhanced CT revealed that the leiomyoma was closely adjacent to the heart and major vessels, indicating a high risk of major intraoperative bleeding (Figure 3). Therefore, combined surgical removal of both lesions was considered technically highly challenging, highlighting potential intraoperative risks.

After careful consideration of the oncological safety, procedural risks, and the patient’s strong preference for esophageal preservation, a minimally invasive approach was favoured. Shared decision-making between the clinical team and the patient led to the decision to proceed with selective endoscopic submucosal dissection (ESD) to achieve complete resection of the superficial carcinoma, with planned endoscopic surveillance of the leiomyoma.

**Figure 4:**
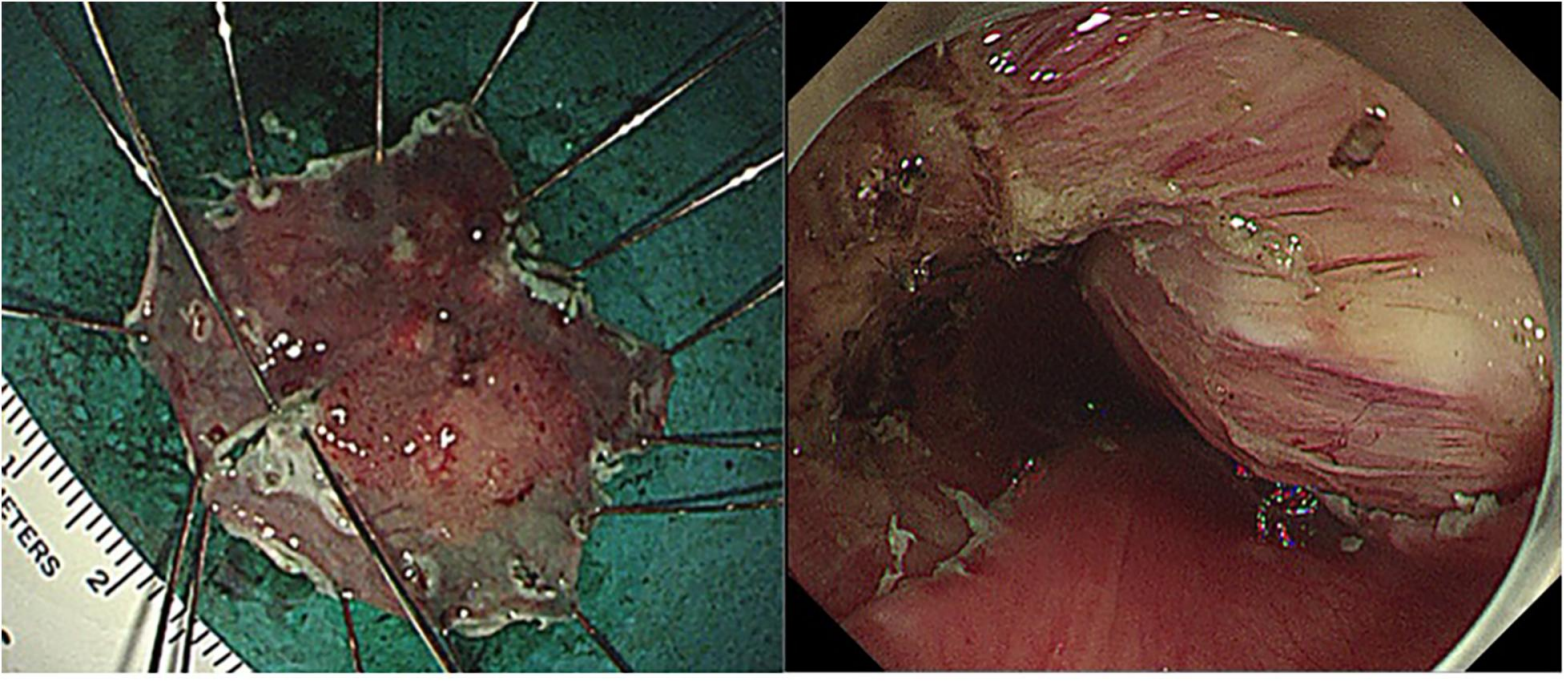
Outcome following selective endoscopic submucosal dissection. Endoscopic view following selective endoscopic submucosal dissection, showing *en bloc* resection of the superficial carcinoma with preservation of the underlying leiomyoma.

ESD was performed under endoscopic and EUS guidance. The lesion was carefully marked, and a submucosal injection of glycerol solution was administered to achieve adequate mucosal elevation. Dissection was carried out meticulously along the submucosal plane, allowing *en bloc* resection of the neoplastic lesion while preserving the integrity of the underlying leiomyoma. Despite the large size of the leiomyoma and its proximity to the muscularis propria, the procedure was completed successfully without intraoperative complications (Figure 4). Adequate haemostasis was achieved, and the resected specimen was retrieved for histopathological examination.

Histopathological analysis revealed a well-differentiated superficial ESCC measuring 2.3 × 2.5 cm. The tumor invaded the submucosal layer with a depth greater than 200 μm (pT1b). Both horizontal and vertical margins were negative (R0 resection). No lymphovascular invasion was identified. The discrepancies between biopsy findings and final pathology may be attributed to the limited sampling depth of endoscopic biopsy, which may underestimate the true depth of invasion, particularly in lesions with submucosal involvement.

After postoperative pathology confirmed pT1b disease, additional surgical treatment was recommended because of the risk of lymph node metastasis; however, the patient strongly refused. Contrast-enhanced CT showed no evidence of regional lymph node or distant metastasis, and the patient subsequently underwent radiotherapy at a local hospital. Follow-up at 1 month, 6 months, and 2 years showed no recurrence, and the leiomyoma remained stable in size and morphology. Detailed follow-up information is summarized in Supplementary Table 1.

## 3 Discussion

### 3.1 Diagnostic pitfall: malignancy concealed by a benign-appearing lesion

Superficial esophageal squamous cell carcinoma (ESCC), including lesions with submucosal invasion, is well suited to minimally invasive treatments such as endoscopic submucosal dissection (ESD), which allows *en bloc* resection while preserving esophageal structure and function. However, the coexistence of a benign submucosal lesion, such as a leiomyoma, necessitates heightened diagnostic vigilance. Benign-appearing lesions may dominate the endoscopic visual field and obscure subtle mucosal abnormalities, increasing the risk that an underlying malignancy will be overlooked or incorrectly interpreted (3).

This diagnostic imperative is particularly relevant in asymptomatic patients undergoing screening or surveillance endoscopy, in whom clinical suspicion for malignancy may be low. In the present case, the large leiomyoma could easily have been regarded as the sole pathological finding. Careful inspection of the mucosal surface, however, revealed focal irregularity that prompted further evaluation. This highlights the importance of meticulous mucosal assessment overlying and adjacent to submucosal tumours, as even minor surface changes may represent early malignant transformation.

The coexistence of esophageal squamous cell carcinoma (SCC) and leiomyoma is exceedingly rare (4). As a result, there is a risk that superficial carcinoma may be misdiagnosed as benign mucosal change or attributed to mechanical distortion caused by the underlying mass. Our case reinforces the principle that benign esophageal lesions should not preclude thorough evaluation for synchronous malignancy.

### 3.2 Role of advanced endoscopic imaging in early detection

Advanced endoscopic imaging techniques played a pivotal role in establishing the diagnosis in this case. Narrow-band imaging (NBI) with magnification enabled detailed assessment of the mucosal microvascular architecture, revealing irregular intrapapillary capillary loop patterns characteristic of early SCC (5). Lugol’s iodine chromoendoscopy further delineated iodine-unstained areas corresponding to neoplastic epithelium.

These modalities are particularly valuable when conventional white-light endoscopy reveals only subtle or equivocal abnormalities, especially in the context of submucosal tumors where surface changes may be minimal, and can improve diagnostic accuracy (6). Our findings support the routine consideration of such techniques when evaluating esophageal leiomyomas with any degree of mucosal irregularity.

### 3.3 Therapeutic strategy and patient-centred decision-making

The management of coexisting early esophageal carcinoma and leiomyoma presents a therapeutic dilemma. While surgical resection remains a definitive treatment option, it is associated with considerable morbidity and potential long-term functional impairment. Treatment strategies for superficial esophageal carcinoma have long emphasised the need to balance oncological radicality with preservation of esophageal structure and function, particularly in early-stage disease (7). In contrast, ESD offers a minimally invasive approach that can achieve complete local resection; however, for lesions with submucosal invasion (pT1b), additional treatment is often required due to the risk of lymph node metastasis.

In the present case, the decision to pursue ESD was guided not only by oncological considerations but also by patient preference and quality-of-life concerns. The patient expressed apprehension regarding the potential complications of surgery, including postoperative reflux and its impact on daily living. Shared decision-making between the multidisciplinary team and the patient was central to selecting an organ-preserving approach (8).

In this case, several lesion-specific factors supported a conservative approach for the leiomyoma. The tumor was relatively large (4.4 cm) and originated from the muscularis propria, with close anatomical proximity to critical mediastinal structures, including the aorta. These features were considered to increase the technical difficulty and procedural risks of resection, including bleeding, perforation, and potential mediastinal complications, particularly in larger lesions arising from the muscularis propria (9, 10). Although advanced endoscopic techniques such as submucosal tunneling endoscopic resection (STER) were considered, preoperative imaging showed that the tumor’s adjacency to the heart and major vessels posed a high risk of massive bleeding, and endoscopic removal was considered technically very challenging (10, 11). Therefore, selective resection of the superficial carcinoma with observation of the leiomyoma was considered the safest approach.

In addition, the leiomyoma was asymptomatic and showed no high-risk features on imaging or endoscopic evaluation. After multidisciplinary discussion, the management options, including endoscopic or surgical resection vs. surveillance, were carefully explained to the patient and her family. Given the benign nature of the lesion and the potential procedural risks, the patient and her family preferred a conservative approach with regular follow-up showing no recurrence and stable leiomyoma size.

Herein, we report a complete ESD for superficial ESCC with submucosal invasion, with successful preservation of a coexisting large leiomyoma. This case highlights the importance of careful evaluation of seemingly benign esophageal lesions, as subtle mucosal abnormalities may conceal early malignancy. A meticulous endoscopic assessment enabled the detection of the carcinoma despite the overlying leiomyoma, allowing targeted intervention while preserving the underlying benign tumor.

Compared with previously reported cases summarized in Supplementary Table 2, most studies involved smaller leiomyomas or management strategies in which both the epithelial lesion and the submucosal tumor were resected, either endoscopically or surgically. In contrast, our case involved a relatively large leiomyoma (4.4 cm) arising from the muscularis propria that was intentionally preserved. A selective ESD approach was employed to achieve complete treatment of the superficial carcinoma while maintaining procedural safety and functional integrity. This individualized strategy, guided by lesion characteristics, anatomical considerations, and patient preference, represents a minimally invasive management paradigm for selected cases of coexisting malignant and benign esophageal lesions.

For comparison, only cases with clearly documented superficial esophageal squamous neoplasia coexisting with leiomyoma and detailed management information were included in Supplementary Table 2. Earlier reports have described similar coexistence; however, many predate modern endoscopic techniques or involve heterogeneous clinical scenarios, and were therefore excluded from the comparative analysis.

### 3.4 Technical considerations and procedural feasibility

Technical challenges during ESD in the presence of a leiomyoma include achieving adequate mucosal elevation over the underlying mass and avoiding injury to the muscularis propria. In this case, careful submucosal injection and meticulous dissection along the appropriate tissue plane were essential to ensure safe *en bloc* resection. Advanced endoscopic techniques, coupled with thorough pre-procedural planning, enabled successful completion of the procedure without complications.

Previous reports have described similar cases in which endoscopic resection was successfully employed to treat superficial SCC coexisting with leiomyoma, with favourable outcomes and minimal procedural morbidity (2, 12, 13). These reports, together with our findings, suggest that ESD is technically feasible and safe in carefully selected patients, even when large submucosal tumours are present (14).

### 3.5 Follow-Up and long-term considerations

Long-term follow-up remains an important consideration in such cases. Although esophageal leiomyomas are generally benign, they may enlarge over time and, in rare instances, undergo malignant transformation (15). Surgical resection may be warranted in cases of symptomatic progression or rapid growth. However, for asymptomatic patients, rigorous endoscopic surveillance represents a reasonable and less invasive alternative.

In this case, follow-up at 1 month, 6 months, and 2 years showed no recurrence and stable leiomyoma size. Given that pT1b ESCC carries a risk of lymph node metastasis, additional surgical treatment was recommended after postoperative pathology; however, the patient strongly declined. Contrast-enhanced CT showed no evidence of metastasis, and the patient subsequently underwent radiotherapy. The favorable 2-year outcome should therefore be interpreted in the context of individualized multidisciplinary management rather than endoscopic treatment alone, supporting a tailored strategy in selected patients when surgery is declined and close follow-up is feasible (9).

Overall, this case supports a tailored, minimally invasive approach to complex esophageal pathology, highlighting the role of advanced endoscopic techniques and patient-centred decision-making in achieving optimal clinical outcomes.

### 3.6 Strengths and limitations

The strength of this case lies in the successful organ-preserving management of a rare coexistence of superficial carcinoma and a large submucosal tumor. However, as a single case report, generalizability remains limited. In addition, although 2-year follow-up showed no recurrence and no enlargement of the leiomyoma, longer-term surveillance is still warranted.

### 3.7 Patient perspective

The patient expressed strong preference for esophageal preservation and was concerned about the potential morbidity and long-term impact of surgical resection. She was satisfied with the minimally invasive approach and reported no postoperative discomfort or functional impairment during follow-up. She expressed appreciation for the shared decision-making process and the individualized treatment strategy. She reported relief knowing that radical surgery could be avoided.

## 4 Conclusion

The rare coexistence of esophageal squamous cell carcinoma (SCC) and leiomyoma poses unique diagnostic and therapeutic challenges. This case highlights the critical need for early vigilance when evaluating benign-appearing esophageal lesions, as submucosal tumors may obscure subtle mucosal abnormalities and delay the recognition of underlying malignancy. Careful inspection of the mucosal surface, supported by advanced endoscopic imaging techniques, is essential to avoid missed or delayed diagnoses, particularly in asymptomatic patients undergoing screening examinations.

The successful use of endoscopic submucosal dissection (ESD) in this case demonstrates its efficacy and favourable therapeutic outcome in the treatment of early-stage SCC while preserving the underlying benign tumor. Selective resection allowed complete local removal of the esophageal lesion while preserving the underlying benign tumor.

Importantly, this case underscores the value of individualized treatment planning. Incorporating clinical findings, procedural feasibility, and patient preferences through shared decision-making is essential for achieving optimal oncological and functional outcomes in complex and rare clinical scenarios.

## Data Availability

The original contributions presented in the study are included in the article/Supplementary Material, further inquiries can be directed to the corresponding authors.
