# Supplementary material for: Case Report: Complete endoscopic submucosal dissection for occult superficial esophageal squamous cell carcinoma concealed by a large esophageal leiomyoma
Source: Front Surg. 2026 May 20;13:1819413. doi: 10.3389/fsurg.2026.1819413 (PMC13229820; doi:10.3389/fsurg.2026.1819413)
Supplement: Supplementary file 1 [file Table1.docx]

Supplementary Material

# Supplementary Table S1. Timeline of the patient’s diagnosis, treatment, and follow-up

| Time Point | Event Description |
| --- | --- |
| Pre-Hospitalization (3 months before) | A 4.4 × 2.5 cm submucosal mass in the mid-esophagus was identified during routine screening, suspected to be a benign esophageal leiomyoma. |
| \| **Day 0 (Admission)** \| \| --- \|  \|  \| \| --- \| | \| Upper gastrointestinal endoscopy revealed a prominent submucosal bulge with focal mucosal irregularity, raising suspicion of a coexisting superficial esophageal squamous cell carcinoma. \| \| --- \|  \|  \| \| --- \| |
| Day 1 (Pre-Operative) | Endoscopic ultrasonography (EUS) and narrow-band imaging (NBI) confirmed a leiomyoma with focal mucosal thickening. Targeted biopsies revealed carcinoma in situ. |
| Day 2 (Multidisciplinary Discussion) | A multidisciplinary team (MDT) recommended selective endoscopic submucosal dissection (ESD) to remove the carcinoma while preserving the leiomyoma. |
| Day 4 (Procedure) | Selective ESD was successfully performed with en bloc resection of the carcinoma and preservation of the underlying leiomyoma, without complications. |
| Day 5 (Postoperative) | The postoperative course was uneventful. Histopathology confirmed well-differentiated ESCC with submucosal invasion (>200 μm, pT1b) and negative margins. |
| Postoperative reassessment | After pathology confirmed pT1b disease, additional surgery was recommended but declined. Contrast-enhanced CT showed no metastasis, and the patient subsequently underwent radiotherapy. |
| 1-Month Follow-up | Follow-up showed no recurrence and stable leiomyoma size. |
| 6-Month Follow-up | Follow-up showed no recurrence and stable leiomyoma size. |
| 2-Year Follow-up | Follow-up showed no recurrence and stable leiomyoma size. |

# Supplementary Table S2. Reported cases of superficial esophageal squamous neoplasia coexisting with esophageal leiomyoma and their management strategies

| **Author (Year)** | **Reference No.** | **Leiomyoma size** | **Origin layer** | **Location** | **Neoplasia depth** | **Treatment for SCC** | **Leiomyoma management** | **Outcome** |
| --- | --- | --- | --- | --- | --- | --- | --- | --- |
| Nagashima et al. (1997) | [12] | Not reported | Not reported | Upper esophagus | Superficial carcinoma | Endoscopic resection | Removed (en bloc) | No recurrence reported (short-term) |
| Mizobuchi et al. (2004) | [1] | Not reported | Not reported | Upper esophagus | Early carcinoma | Surgical resection | Removed | Not reported |
| Niimi et al. (2009) | [2] | 4.2 × 2.6 mm (muscularis mucosae) | Muscularis mucosae | Middle thoracic esophagus | Intraepithelial carcinoma | ESD (en bloc) | Removed (en bloc with lesion) | No recurrence reported |
| Ishida et al. (2013) | [13] | 5 × 4 mm | Muscularis mucosae | Not specified | SCC in situ | Endoscopic resection | Removed | Not reported |
| Liang et al. (2020) | [14] | Not reported | Muscularis propria | Upper esophagus | Early carcinoma | ESD | Removed | No recurrence at 6 months |
| Guo et al. (2021) | [3] | Variable (case series) | Muscularis mucosae / propria | Various | Intramucosal/submucosal ESCC or HGD (case series) | ESD in all 12 cases | Mixed (removed or preserved) | No recurrence during follow-up |
| Present case | — | 4.4 × 2.5 cm | Muscularis propria | Mid esophagus | Submucosal invasive SCC (pT1b) | Selective ESD followed by radiotherapy | Preserved (surveillance) | No recurrence at 2 years after ESD and subsequent radiotherapy; leiomyoma stable |
